# Generation of mouse lines with conditionally or constitutively inactivated *Snca* gene and *Rosa26*-*stop*-*lacZ* reporter located *in cis* on the mouse chromosome 6

**DOI:** 10.1007/s11248-016-9995-8

**Published:** 2016-11-12

**Authors:** Andrei Yu. Roman, Galina Limorenko, Alexey A. Ustyugov, Tatiana V. Tarasova, Ekaterina A. Lysikova, Vladimir L. Buchman, Natalia Ninkina

**Affiliations:** 1 Institute of Physiologically Active Compounds, Russian Academy of Sciences, 1 Severniy proezd, Chernogolovka, Moscow Region Russian Federation 142432; 2 Inserm, CRO2 UMR_S 911, Faculté de Pharmacie, Aix-Marseille Université, 13385 Marseille, France; 3 School of Biosciences, Cardiff University, Museum Avenue, Cardiff, CF10 3AX UK

**Keywords:** Synuclein, Conditional gene knockout, Meiotic recombination, Transgenic mice, LoxP/Cre recombination

## Abstract

α-Synuclein is involved in many important molecular processes in neuronal cells and their synapses, and its malfunction has been linked to the development of Parkinson’s and certain other neurodegenerative diseases. Animal models allowing tightly monitored conditional inactivation of the encoding gene, *Snca*, are indispensible for studies aimed at understanding normal function of α-synuclein in various neuronal populations and its role in pathogenesis of neurodegenerative diseases. We have recently reported the production of several novel mouse lines for manipulating expression of the endogenous *Snca* gene, including a line for Cre-recombinase-driven conditional inactivation of the gene (mice with floxed *Snca*) and a new line with a constitutive knockout of α-synuclein. *Rosa26*-*stop*-*lacZ* reporter cassette is commonly used for monitoring efficiency of Cre-recombination but in mouse genome *Snca* and *Rosa26* loci are located on the same chromosome. Here we describe production of lines with a modified *Snca* locus, either floxed or constitutively inactivated and the *Rosa26*-*stop*-*lacZ* reporter cassette located *in cis* on the mouse chromosome 6. These new mouse lines are invaluable for fast identification of cells with inactivation of *Snca* by Cre-recombination and represent useful tools for in vivo studies of α-synuclein function and dysfunction.

## Introduction

Several missense mutations altering physico-chemical properties of α-synuclein as well as increased production of α-synuclein caused by duplications and triplications of the genomic region containing encoding gene, *SNCA*, have been associated with the development of familial forms of Parkinson’s disease (PD) and related disorders (Polymeropoulos et al. 1997; Kruger et al. 1998; Singleton et al. 2003; Zarranz et al. 2004; Chartier-Harlin et al. 2004; Ibanez et al. 2004; Proukakis et al. 2013; Appel-Cresswell et al. 2013; Kiely et al. 2013; Lesage et al. 2013). Moreover, Lewy bodies and Lewy neurites, histopathological hallmarks of both hereditary and idiopathic forms of PD, are built on a scaffold of aggregated and fibrillated α-synuclein (Spillantini et al. 1997, 1998). Genome-wide associated and case–control studies also linked α-synuclein to idiopathic and familial forms of PD as well as other synucleinopathies (Kay et al. 2008; Mizuta et al. 2008; Pankratz et al. 2009; Scholz et al. 2009; Sutherland et al. 2009).

It is still not clear how altered α-synuclein metabolism triggers the development of pathological changes in these neurodegenerative diseases. The gain-of-function hypothesis is based on multiple evidence of the toxicity of intermediate products of α-synuclein aggregation, oligomers and protofibrils. However, α-synuclein is important for structural and functional integrity of vertebrate neurons and/or their synapses (reviewed in Venda et al. 2010) and pathological aggregation of this protein might cause significant depletion of its functional pool in neurons and particularly in presynaptic terminals. Therefore, a contribution of α-synuclein loss-of-function mechanism to molecular pathogenesis of synucleinopathies cannot be discounted, despite no obvious signs of neurodegeneration have been observed in studies of constitutive α-synuclein knockout mice [reviewed in Buchman and Ninkina (2008)]. These discouraging results can be explained by efficient mechanisms compensating to the loss of α-synuclein. It has been suggested that this compensation takes place in the developing nervous system during a period of its high plasticity (Al-Wandi et al. 2010). Depletion of α-synuclein in adult or ageing nervous system might be significantly more deleterious. To test this hypothesis an experimental system allowing conditional inactivation of α-synuclein encoding gene is required. Moreover, such system would be invaluable for assessing α-synuclein function in specific neuronal populations as well as in studies of α-synuclein aggregation pathology propagation through the nervous system.

Conditional gene knockout in mice requires a core mouse line with the gene of interest or its fragment surrounded by recognition sites for certain specific recombinases, most commonly Cre or FRT, and a transgenic mouse line expressing this recombinase in the particular type of cells. It is important to monitor the efficiency and specificity of recombination and to achieve this a reporter gene whose activation by a recombination event driven by the same enzyme can be easily detected, is also introduced in the animal genome. For monitoring Cre-loxP recombination a *Rosa26*-*stop*-*lacZ* reporter cassette is widely used. This reporter is usually delivered by another mouse line carrying a loxP-flanked transcription STOP sequence, which prevents expression of the downstream bacterial *lacZ* gene encoding β-galactosidase (Soriano 1999). Activation of Cre-recombinase in a cell carrying both the reporter and the floxed gene of interest leads to simultaneous deletion of both floxed genomic fragments leading to inactivation of the studied gene and activation of *lacZ* expression, the latter can be readily detected due to accumulation of a colored product of β-galactosidase activity in cells when specific substrates are added to tissue samples.

We intended to use the *Rosa26*-*stop*-*lacZ* reporter to monitor conditional inactivation of α-synuclein coding gene (*Snca*) in mice carrying this gene with loxP-flanked first coding exon [*Snca* ^*flox*^] (Ninkina et al. 2015) and tamoxifen-activated Cre-recombinase (Cre/ERT2) under control of various neuron-specific promoters but this was obscured by co-localisation of *Snca* and *ROSA26* loci on the same mouse chromosome 6. To overcome this obstacle we have previously produced and used a mouse line with *Rosa26*-*stop*-*lacZ* cassette and permanently inactivated *Snca* gene (Abeliovich et al. 2000) located on the same chromosome *in cis*. Here we describe a similar line but with a version of permanent *Snca* gene knockout that lack problems associated with the presence of *Neo* cassette in the genome of mice produced by Abeliovich et al. (2000), for example dramatic overexpression of Mmrn1 gene (Ninkina et al. 2015). We also produced a mouse line that carries *Snca* ^*flox*^ gene and *Rosa26*-*stop*-*lacZ* reporter cassette located *in cis* at the mouse chromosome 6. These lines represent useful tools for production of large cohorts of experimental animals for conditional inactivation of α-synuclein function.

## Materials and methods

### Animals

All animals were on C57Bl6J genetic background. Animal work was carried out in accordance with the United Kingdom (Scientific Procedures) Act (1986) and European Directive EC 86/609, and has been approved by the Cardiff University Ethical Review Committee and the Home Office (Project Licence 30/2844). The core mouse line for conditional inactivation of *Snca* gene, [*Snca* ^*flox*^ *_Rosa26* ^*wt*^/*Snca* ^+^ *_Rosa26* ^*wt*^], has been deposited to and now available from The Jackson Laboratory (C57BL/6-Snca<tm1.1Vlb>/J; JAX Stock#025636).

### Genotyping

Genomic DNA was isolated from mouse ear biopsies as described elsewhere (Ninkina et al. 2009). PCR amplification was employed to verify genotypes.

For *Rosa26* locus combination of three primers in the reaction (5′-CTTGTGATCCGCCTCGGAGT-3′; 5′-GGCATTCATGGGAGTGGAAA-3′; 5′-TACTGGCCTGCTCCCTTATC-3′) produced 577 and 450 bp amplification fragments corresponding to wild type and modified alleles, respectively.

For *Snca* locus combination of three primers in the reaction (5′-TGCTGGGCACAGTGTTGATTG-3′; 5′-AAAGGCTGGGCTTCAAGCAG-3′; 5′-CATGAGTACTTGTGGCTCAC-3′) produced amplification fragments of 354 bp for *Snca* ^+^, 280 bp for *Snca* ^−^ and 406 bp for *Snca* ^*flox*^ alleles, respectively.

For both amplification reactions 94 °C for 2 min followed by 45 cycles of 94 °C for 15 s, 60 °C for 20 s and 72 °C for 30 s were used.

### Western blot analysis

Total protein extraction from mouse neuronal tissues, SDS-PAGE separation, semi-dry transfer, blocking, incubation with antibodies, washing and detection using enhanced chemiluminescence were carried out as described previously (Anwar et al. 2011). Antibodies against α-synuclein (mouse monoclonal, clone 42, BD Transduction Laboratories, diluted 1:500) and β-actin (mouse monoclonal, clone AC-15, Sigma diluted 1:5000) were used.

### Detection of β-galactosidase activity in brain slices

[*Snca* ^*flox*^ *_Rosa26* ^*mod*^/*Snca* ^*flox*^ *_Rosa26* ^*mod*^] mice were crossed with transgenic mice expressing Cre-ERT2 under control of neurospecific NSE promoter (obtained from Jean C. Manson, The Roslin Institute, University of Edinburgh) and at the age of 3 month resulting F1 animals were injected intraperitoneally with tamoxifen (Sigma-Aldrich) dissolved in corn oil for five consecutive days (daily dose 75 mg/kg). One month after activation of Cre recombination by tamoxifen brains were dissected and transverse slices (~1 mm thick) through the brainstem were processed and stained for β-galactosidase activity using X-gal as a substrate as described elsewhere (Burn 2012).

## Results and discussion

**Fig. 1:**
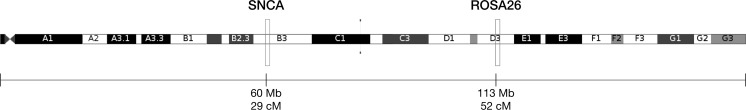
Relative positions of *Snca* and *Rosa26* loci on mouse chromosome 6

Within our previous breeding programme a mouse founder has been obtained that carried a permanently inactivated *Snca* locus from mice originally produced by Abeliovich et al. (2000) and *Rosa26*-*stop*-*lacZ* cassette (Soriano 1999) on the same chromosome 6 *in cis*. As the physical distance between *Snca* and *Rosa26* loci is 52.3 Mb, which constitutes more than one-third of total 150 Mb length of mouse chromosome 6 (Fig. 1), and genetic distance is 23.5 cM, high meiotic recombination frequency between these loci is expected. Therefore we decided to carry out additional breeding programme aimed for obtaining mouse lines carrying other genetic modifications of *Snca* gene *in cis* with *Rosa26*-*stop*-*lacZ* reporter cassette.

**Fig. 2:**
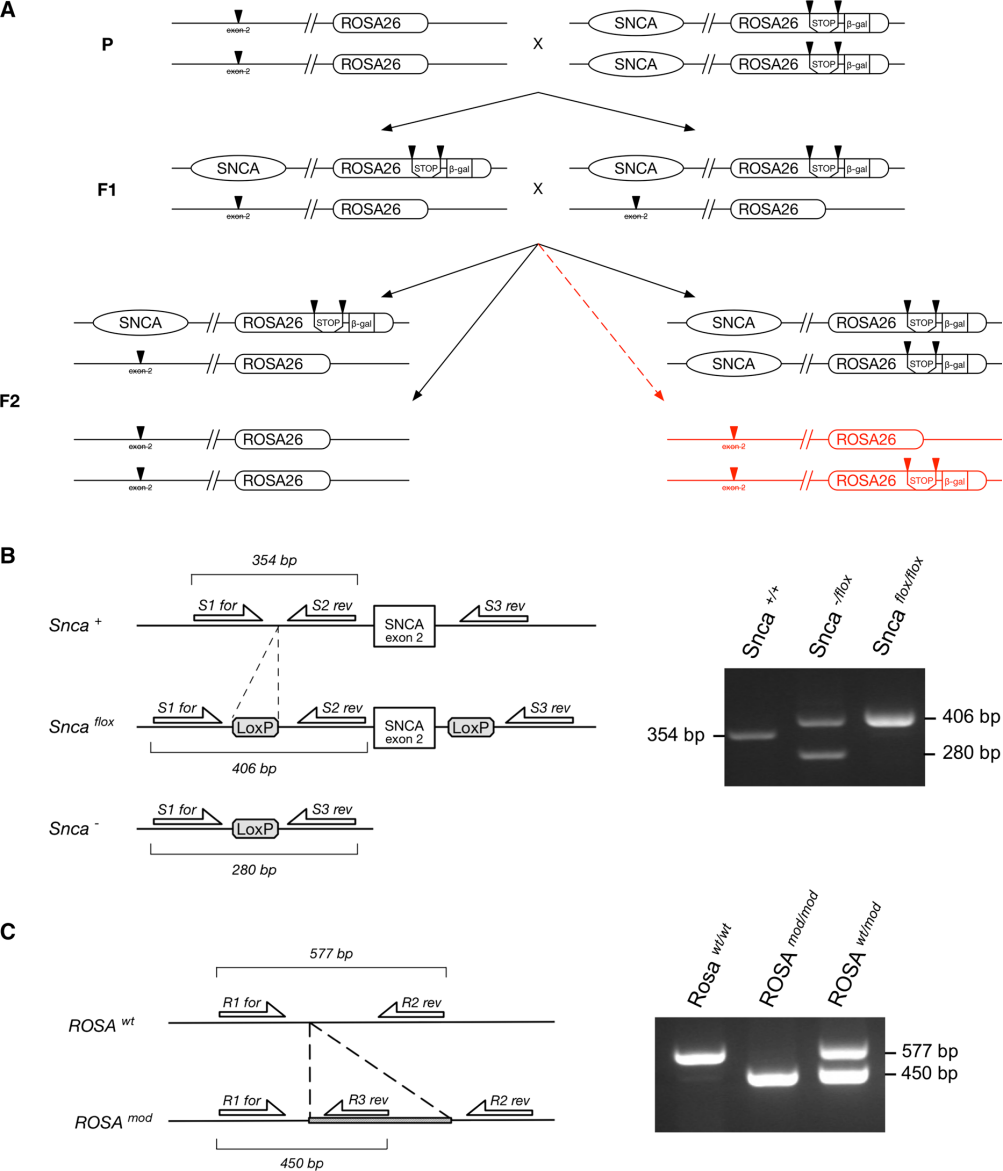
A sheme of the breeding protocol that produced mouse founders carrying a constituent knock-out of α-synuclein-encoding gene  and *Rosa26*-*stop*-*lacZ* cassette located *in cis* on mouse chromosome 6 as the result of meiotic recombination (**a**). PCR amplification approach used for identification of modifications in *Snca* (**b**) and *Rosa26* (**c**) loci. Position of primers within these loci and analysis of corresponding amplification products in agarose gel are shown

The deletion of the *Snca* exon 2 (first coding exon) by Cre-loxP recombination in the germline causes complete arrest of α-synuclein production in homozygous *Snca* ^−^/*Snca* ^−^ mice. This genomic modification does not affect expression of neighboring genes, e.g. Mmrn1 (Ninkina et al. 2015), which is a drawback for the knockout mice produced by Abeliovich et al. Mice homozygous for this Cre-induced deletion, [*Snca* ^−^ *_Rosa26* ^*wt*^/*Snca* ^−^ *_Rosa26* ^*wt*^], were crossed with homozygous mice bearing *Rosa26*-*stop*-*lacZ* cassette, [*Snca* ^+^ *_Rosa26* ^*mod*^/*Snca* ^+^ *_Rosa26* ^*mod*^], and resulting F1 offsprings of [*Snca* ^+^ *_Rosa26* ^*mod*^/*Snca* ^−^ *_Rosa26* ^*wt*^] genotype were intercrossed (Fig. 2a). F2 offsprings were genotyped for both loci (Fig. 2b, c). Between 26 analysed animals one displayed genotype [*Snca* ^−^ *_Rosa26* ^*mod*^/*Snca* ^−^ *_Rosa26* ^*wt*^] (shown in red in Fig. 2a), which was not consistent with classical Mendelian inheritance that predicts segregation of chromosomes and independent assortment of alleles. Most probably this genotype appeared as a result of a reciprocal recombination between chromosome 6 bearing *Rosa26*-*stop*-*lacZ* cassette and chromosome 6 bearing *Snca* null mutant gene during meiosis I phase of gametogenesis leading to non-Mendelian segregation of traits. Consequently, a male mouse that became a founder of a new line carried a chromosome 6 with both *Snca* ^−^ and *Rosa26* ^*mod*^ loci *in cis*. Further backcrossing and intercrossing produced a line of homozygous [*Snca* ^−^ *_Rosa26* ^*mod*^/*Snca* ^−^ *_Rosa26* ^*mod*^] mice.

**Fig. 3:**
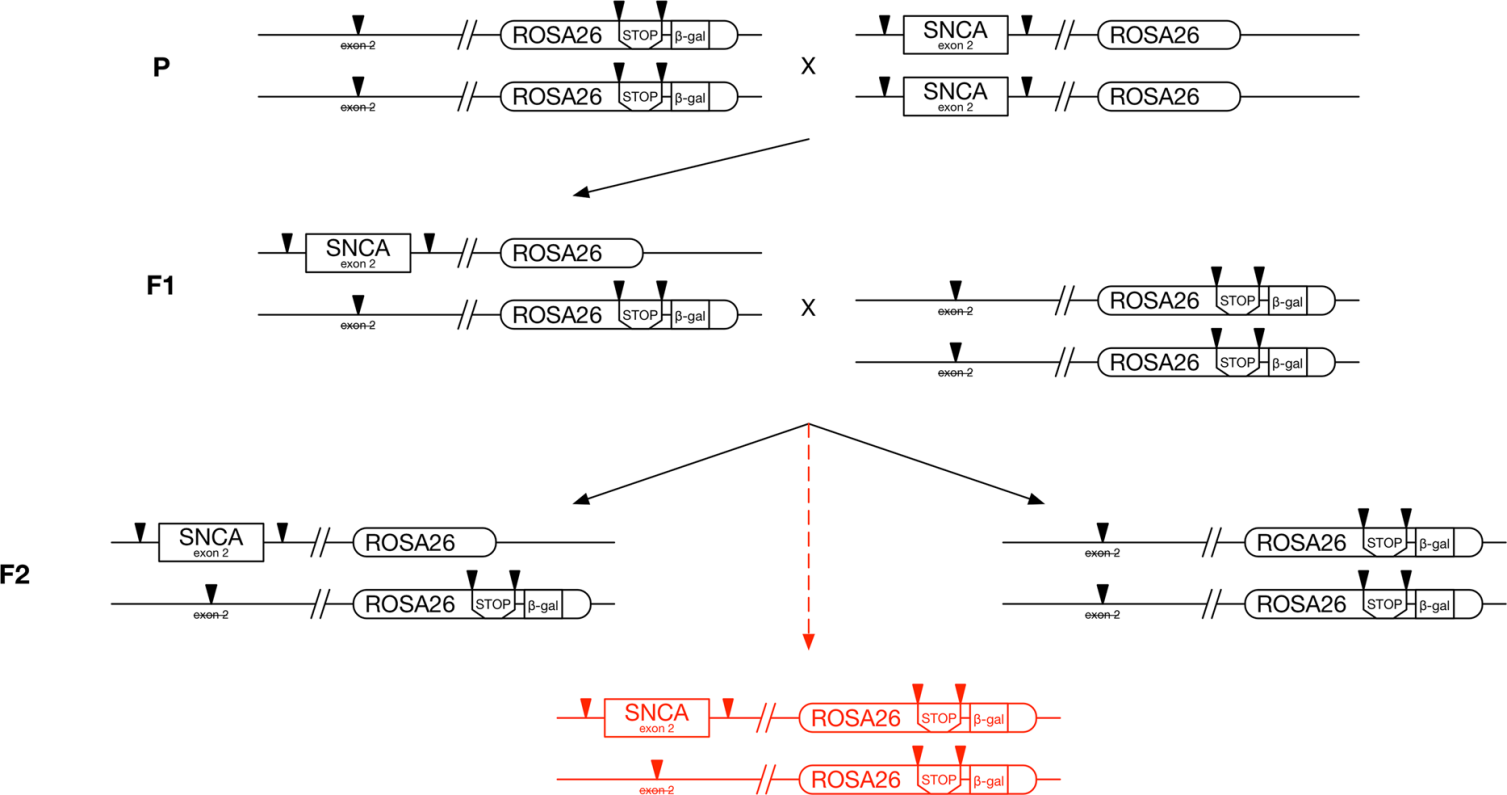
A sheme of the breeding protocol that produced mouse founders carrying floxed exon 2 of α-synuclein-encoding gene  and *Rosa26*-*stop*-*lacZ* cassette located *in cis* on mouse chromosome 6 as the result of meiotic recombination

These homozygous mice were further crossed with homozygous mice of the core line for conditional inactivation of α-synuclein-encoding gene, [*Snca* ^*flox*^/*Snca* ^*flox*^], that have the second exon of *Snca* gene flanked with loxP sites in direct orientation. F1 offspring heterozygous for both *Snca* and *Rosa26* loci, [*Snca* ^−^ *_Rosa26* ^*mod*^/*Snca* ^*flox*^ *_Rosa26* ^*wt*^], were crossed again with homozygous [*Snca* ^−^ *_Rosa26* ^*mod*^/*Snca* ^−^ *_Rosa26* ^*mod*^], the *Rosa26* genotype of the offspring was analysed and animals carrying *Rosa26*-*stop*-*lacZ* cassette on both chromosomes were selected for further analysis. *Snca* genotypes were assessed for 130 of these F2 mice and although majority of animals displayed either of two expected Mendelian genotypes, a [*Snca* ^−^ *_Rosa26* ^*mod*^/*Snca* ^*flox*^ *_Rosa26* ^*mod*^] genotype (shown in red in Fig. 3), was revealed in 11 animals.

Meiotic recombination frequency between these two loci seems higher during oogenesis than during spermatogenesis as crosses that involved female F1 [*Snca* ^−^ *_Rosa26* ^*mod*^/*Snca* ^*flox*^ *_Rosa26* ^*wt*^] parent produces 7 F2 [*Snca* ^−^ *_Rosa26* ^*mod*^/*Snca* ^*flox*^ *_Rosa26* ^*mod*^] animals from total 64 homozygous *Rosa26* ^*mod*^ animals, whereas for crosses that involved male F1 [*Snca* ^−^ *_Rosa26* ^*mod*^/*Snca* ^*flox*^ *_Rosa26* ^*wt*^] parent these numbers were 4 from 67. Although these results are not statistically significant with the number of animals assessed (*p* > 0.05, Chi-square test) it might still be taken into consideration when experimental cohorts are generated using a breeding programme with meiotic recombination between these loci is possible but not desired.

Male and female [*Snca* ^−^ *_Rosa26* ^*mod*^/*Snca* ^*flox*^ *_Rosa26* ^*mod*^] founders were intercrossed to produce homozygous [*Snca* ^*flox*^ *_Rosa26* ^*mod*^/*Snca* ^*flox*^ *_Rosa26* ^*mod*^] animals that were further used for establish a new mouse line.

**Fig. 4:**
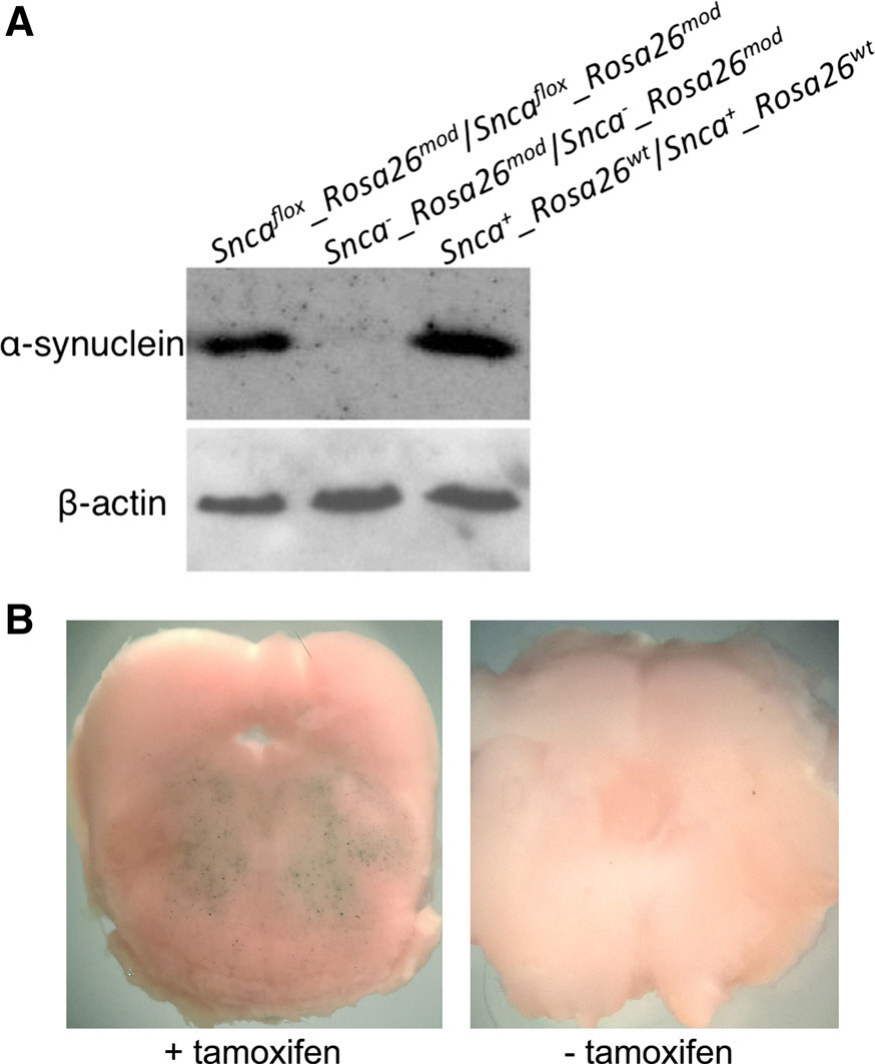
Western blot analysis of α-synuclein expression in the brainstem of mice obtained in this study (**a**) and detection of β-galactosidase activity using X-gal staining in transverse slices through the brainstem of [*Snca* ^*flox*^ *_Rosa26* ^*mod*^/*Snca* ^*flox*^ *_Rosa26* ^*mod*^] mice following tamoxifen-induced activation of Cre-ERT2 recombinase in neurons (**b**)

In this mouse line expression of α-synuclein in neuronal tissues was the same as in wild type, [*Snca* ^+^ *_Rosa26* ^*wt*^/*Snca* ^+^ *_Rosa26* ^*wt*^], animals, whereas no α-synuclein was detected in neuronal tissues of the new knockout, [*Snca* ^−^ *_Rosa26* ^*mod*^/*Snca* ^−^ *_Rosa26* ^*mod*^], mice (Fig. 4a). As expected, activation of Cre-recombinase in neurons of [*Snca* ^*flox*^ *_Rosa26* ^*mod*^/*Snca* ^*flox*^ *_Rosa26* ^*mod*^] mice led to expression of *lacZ* gene from the modified *Rosa26* locus (Fig. 4b).

Two new transgenic mouse lines produced in this study complement a set of previously described mouse lines (Ninkina et al. 2015) for manipulating endogenous α-synuclein expression and makes generation of experimental cohorts for experiments required conditional inactivation of α-synuclein function relatively easy and straightforward.

For example, it is sufficient to obtain mice carrying a copy of relevant Cre-recombinase along with a copy of *Snca* ^*flox*^ allele, i.e. [*Snca* ^*flox*^ *_Rosa26* ^*wt*^/*Snca* ^+^ *_Rosa26* ^*wt*^], and cross them with [*Snca* ^*flox*^ *_Rosa26* ^*mod*^/*Snca* ^*flox*^ *_Rosa26* ^*mod*^] mice to obtain experimental cohorts, i.e. Cre-positive [*Snca* ^*flox*^ *_Rosa26* ^*mod*^/*Snca* ^*flox*^ *_Rosa26* ^*wt*^] mice, and various control cohorts, i.e. Cre-positive [*Snca* ^*flox*^ *_Rosa26* ^*mod*^/*Snca* ^+^ *_Rosa26* ^*wt*^] mice that will retain one functional copy of *Snca* gene even after Cre-recombination takes place, and Cre-negative mice either homozygous or heterozygous for *Snca* gene. Alternatively, Cre-positive [*Snca* ^*flox*^ *_Rosa26* ^*wt*^/*Snca* ^+^ *_Rosa26* ^*wt*^] mice may be crossed with another line described here, [*Snca* ^−^ *_Rosa26* ^*mod*^/*Snca* ^−^ *_Rosa26* ^*mod*^] mice, to obtain Cre-positive heterozygous *Snca* experimental, [*Snca* ^*flox*^ *_Rosa26* ^*wt*^/*Snca* ^−^ *_Rosa26* ^*mod*^] and Cre-positive heterozygous *Snca* control, [*Snca* ^+^ *_Rosa26* ^*wt*^/*Snca* ^−^ *_Rosa26* ^*mod*^] mice.

Moreover, the [*Snca* ^*flox*^ *_Rosa26* ^*mod*^/*Snca* ^*flox*^ *_Rosa26* ^*mod*^] line is an obvious choice for experiments with non-genetic delivery of Cre-recombinase for local inactivation of *Snca* gene, e.g. stereotaxic injection of recombinant viruses expressing this enzyme, or for preparing primary neuronal cultures and consequent inactivation of *Snca* gene by transfection or viral transduction of Cre-expressing constructs.

In conclusion, we produced two mouse lines that constitute novel useful tools for studying normal function of α-synuclein and its role in pathological processes.
